# Ultrahigh-throughput screening of environmental bacteria for proteolytic activity using droplet-based microfluidics

**DOI:** 10.1128/aem.00109-25

**Published:** 2025-06-13

**Authors:** Akihiro Nakamura, Yoshiyuki Suzuki, Nobuyuki Homma, Yosuke Shida, Rikako Sato, Hiroaki Takaku, Wataru Ogasawara

**Affiliations:** 1 Department of Science of Technology Innovation, Nagaoka University of Technology 52756, Nagaoka-shi, Niigata, Japan; 2 On-chip Biotechnologies Co., Ltd., Koganei-shi, Tokyo, Japan; 3 Department of Materials Science and Bioengineering, Nagaoka University of Technology 52756, Nagaoka-shi, Niigata, Japan; 4 Department of Applied Life Sciences, Niigata University of Pharmacy and Medical and Life Sciences, Akiha-ku, Niigata, Japan; Kyoto University, Kyoto, Japan

**Keywords:** water-in-oil droplet, emulsion, bacterial enzyme, screening

## Abstract

Exploration of diverse microbial sources, particularly environmental bacteria, is needed to identify novel and efficient peptidases/proteases that can be used in a variety of industrial applications. However, conventional function-based screening methods are inefficient and preclude the use of diverse microbial resources. This study illustrates a revolutionary approach to microbial screening using droplet-based microfluidics and fluorescence-activated droplet sorting that targets endopeptidases. Droplet-based microfluidic systems are a powerful tool for culturing microorganisms and for detecting microbial functions inside droplets with ultra-high throughput. However, droplet-based microfluidics for screening the proteolytic activity of environmental bacteria at a large scale remains largely unexplored. Here, we screened approximately 630,000 microorganisms in 6 h and obtained four species with high peptidase activity using droplet-based microfluidics. Furthermore, we isolated an Asp-specific endopeptidase from the isolated bacteria *Lysobacter soli* and showed that its activity was 2.4-fold higher than that of the related commercially available enzyme. The successful isolation of Asp-specific peptidase with superior activity in a short period of time compared to existing alternatives underscores the efficacy of droplet-based microfluidics for function-based microbial screening.

**IMPORTANCE:**

As global efforts to reduce environmental impact progress, realization of the significance of biomanufacturing bio-based products has risen, increasing the demand for microbial-based manufacturing. Producing diverse bio-based products through biomanufacturing requires isolating suitable host organisms from environmental sources and screening them for essential genetic characteristics. Efficient screening methods based on microbial activity and functionality are thus essential to significantly expand the scope of bio-based products. Here, we demonstrate the development of a highly efficient screening system for functional screening of environmental bacteria using a droplet-based microfluidic device. This platform enables the streamlined isolation of microbial strains and acquisition of genetic resources from the environment and is tailored to specific microbial activities and functions. In this study, we have demonstrated the efficacy of this droplet-based method for functional screening and have shown its potential for scalability to industrial levels for advancing bio-based production.

## INTRODUCTION

Environmental bacteria are a vast and underexplored source of enzymes with potential industrial applications (1, 2). In particular, microbial proteolytic enzymes (peptidases or proteases), which catalyze the hydrolysis of peptide bonds, have been harnessed extensively in a multitude of industries, including laundry detergents, food processing, pharmaceuticals, and waste treatment (3). Indeed, the global market for proteases is valued at around USD 2 billion in 2024, demonstrating the scale of their industrial importance (4). Despite the wide utilization of available proteolytic enzymes, the demand for novel and more efficient proteases remains high. Thus, the focus has been exploring diverse microbial sources, especially environmental bacteria, to discover useful new peptidases.

With recent progress in biotechnology, metagenomic library screening using metagenomes obtained from uncultured microorganisms has become popular as a screening method for microbial enzymes (5, 6). Sequence-based metagenomic screening is suitable for finding new gene sequences that reveal similarities to annotated genomes available in databases. However, the sequence-based approach is limited to cases where the function of enzymes with similar sequences is already known. In other words, enzymes with no similar sequence (unknown function) are difficult to obtain using a sequence-based approach. In addition, even if enzyme activity can be predicted from the gene sequence, it is difficult to predict the activity levels, and the enzyme may not have the same activity. This leaves a strong need for function-based screening. The challenge in function-based screening, both in function-based metagenomic screening and in screening host microorganisms at native states, is the throughput of the screening process, which includes the culture and evaluation of microorganisms. Indeed, it has been reported that the number of microorganisms showing target enzyme activity is 10 strains or fewer from a screening of 1 million species (7). Hence, the development of ultrahigh-throughput screening is of paramount importance and could lead to accelerated discovery of industrially relevant enzymes and contribute to expanding the available genetic resources for sequence-based metagenomic approaches.

Droplet-based microfluidic systems using water-in-oil droplets (WODLs) have been explored in ultrahigh-throughput microbial screening methods based on functions (8, 9). In general, these systems encapsulate microbial cells, enzymes, or chemical compounds in discrete, picoliter to nanoliter volume WODLs, allowing for parallel processing of a large number of samples with minimal reagent consumption. The compartmentalization of microbial cells and the miniaturization of microbial cultivation are features specific to this technique, which facilitate the screening of microorganisms according to microbial functions (10, 11). For instance, in microbial screening, culturing of microorganisms and detection of function (enzymes and substances) are conducted at the same time inside nanoliter- or picoliter-sized WODLs, and microorganisms are screened based on function with fluorescence-activated droplet sorting (FADS) (12–15). Currently, environmental microbial screening has been carried out using droplet-based microfluidics systems for antibiotic productivity and for measurement of enzymatic activities such as cellulase and lipase activities (12, 16, 17). Despite these advancements, the use of FADS for screening proteolytic activity from environmental bacteria at a large scale remains largely unexplored (18). Previously, we reported a large-scale screening platform for exopeptidase activity using WODLs from environmental bacteria; however, no microbial enzymes were isolated in this study (19). In addition, the application of this technology to endopeptidases, which are in great industrial demand, is limited.

In this study, we extended the application of the innovative FADS-based microbial ultrahigh-throughput screening system to endopeptidase (protease) activity, which is used in a wide range of industries. In the screening of model microorganisms, we also devised a neat method to calculate turbidity (optical density at 600 nm [OD_600_]), an indicator of growth, from the cell occupancy rate inside the droplets. This method enabled us to verify the behavior of microbial cells in WODL cultures depending on the culture conditions. In our screening using this method, we succeeded in isolating Asp-specific metallo-endopeptidases that showed higher activity than commercially available homologous enzymes used for peptide fingerprinting. It is worth noting that with this system, approximately 630,000 microorganisms were screened based on function in less than 6 h. This study demonstrates the usefulness of droplet-based microfluidic systems for microbial screening based on function.

## RESULTS AND DISCUSSION

### Turbidity measurement in WODL

**Fig 1:**
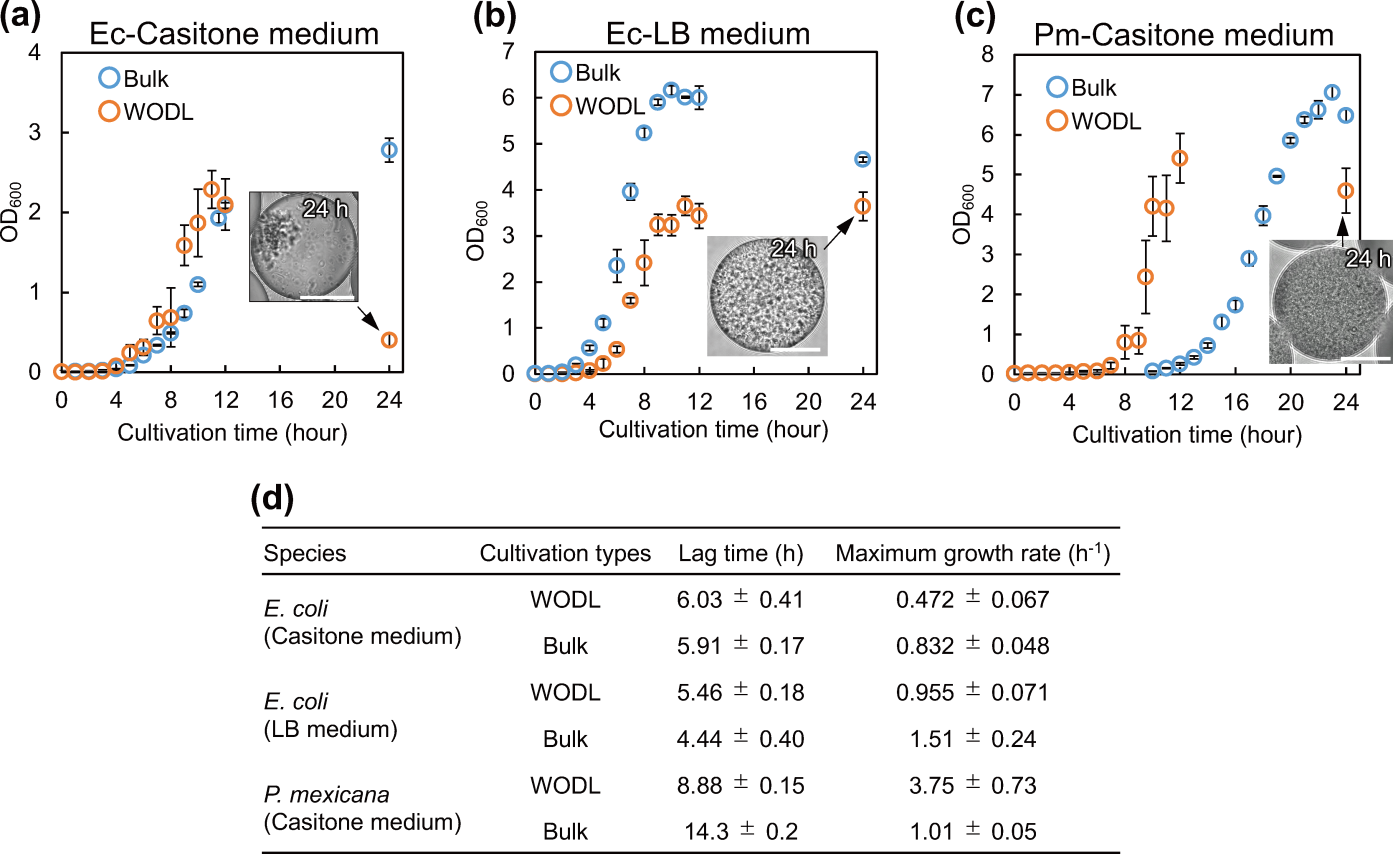
Growth curves of model microorganisms in WODL and bulk cultivation. (a) *E. coli* DH5α was cultivated in casitone medium. (b) *E. coli* DH5α was cultivated in LB medium. (c) *P. mexicana* was cultivated in casitone medium. For bulk cultivation, samples were taken from 50 mL culture medium, and standard deviations were calculated based on three independent measurements of OD_600_. For droplet cultivation, standard deviations were calculated based on five droplets. Inset shows droplet with cultured cells. Droplets containing microorganisms were randomly selected from the imaged set and are presented here. The droplet with cultured cells for the other time points is shown in Fig. S2. The scale bar represents 50 µm. (d) Maximum growth rate and lag time were determined by fitting the experimental data to the growth curve using the Combase database (www.combase.cc).

Turbidity, quantified as OD_600_, is a critical metric for assessing the state of the microorganism culture and to determine whether they are properly cultured as part of the function-based screening process. However, it is difficult to measure the turbidity of droplets in WODL cultures. Understanding how microbial growth in a WODL compares to that in bulk culture (flask culture) or other conditions is paramount for advancing WODL’s application in microbial culture. Here, we introduce a non-destructive method to estimate OD_600_ in WODL using image analysis. Two microbial species, *Pseudoxanthomonas mexicana* WO24 (hereafter *P. mexicana*), known as a high peptidase producer, and *Escherichia coli*, a model microorganism, were employed for validation. To establish the relationship between OD_600_ and droplet occupancy, bulk cultures of each microorganism (with a known OD_600_) were converted into droplets, and their occupancy was determined. The derived calibration curve, with a coefficient of determination exceeding 0.99, signified a high precision (Fig. S1). Subsequently, we encapsulated a single cell in each droplet of both microorganisms and performed microscopic observations at various time points. This allowed us to compute OD_600_ from the occupancy rate (Fig. 1). In *E. coli*, the maximum growth rate in droplet cultivation was lower than that in bulk culture, although the general trends were similar (Fig. 1a). However, after 24 h, *E. coli* cells aggregated within the droplet, leading to a decrease in occupancy compared to the 12 h measurement. This aggregation did not occur in the LB medium, suggesting that it could be specific to casitone medium (Fig. 1b). In contrast, *P. mexicana* demonstrated significant differences between WODL and bulk cultivation (Fig. 1c). The lag time for *P. mexicana* in the WODL was 8.9 h, as opposed to 14.3 h in the bulk culture (Fig. 1d). Furthermore, the maximum growth rate of *P. mexicana* in WODL exceeded that in the bulk culture, suggesting that *P. mexicana* proliferates more rapidly under WODL conditions. This observation indicates that some microorganisms may be better suited for WODL growth than others, necessitating further investigation to elucidate the underlying factors. In conclusion, microbial growth behavior in WODL culture was not always consistent with that in bulk culture, but exhibited species-dependent variability.

Morita et al. employed a comparable technique to evaluate microbial occupancy in liposomes and discovered that *E. coli* in the stationary phase demonstrated a bacterial occupancy rate of approximately 60 ± 13% (20). Our study revealed an occupancy rate of 84 ± 3% after 12 h of *E. coli* droplet culture. It is important to emphasize that the occupancy rate serves as a relative measurement and does not directly represent the actual cell density. By constructing a calibration curve correlating occupancy and OD in our study, we were able to understand different growth behaviors in WODL and bulk cultures.

### Detection and sorting based on endopeptidase activity of bacterial cells

**Fig 2:**
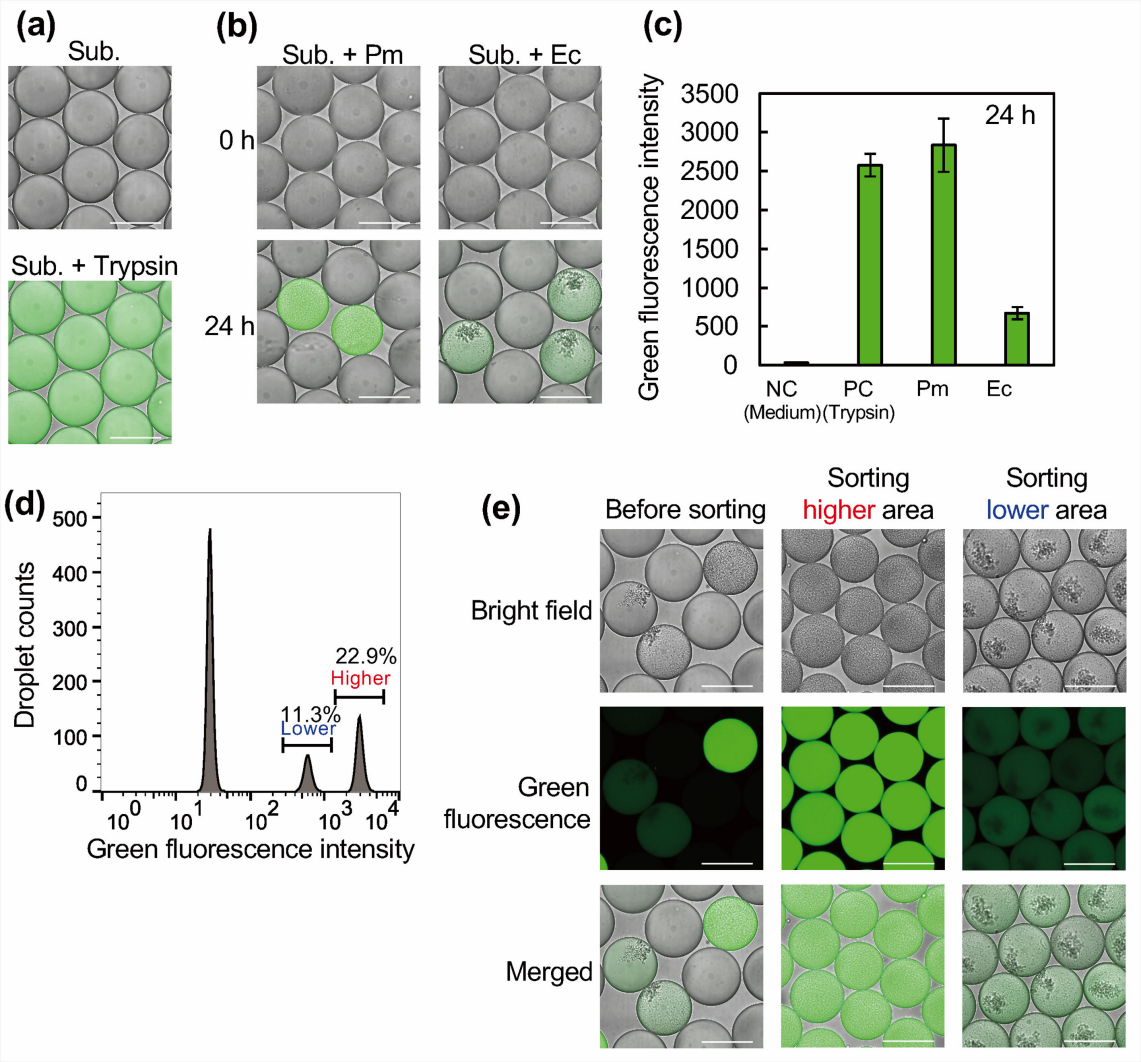
FADS based on endopeptidase activity in model microorganisms. (a) The enzymatic reaction of the fluorescent substrate and trypsin was performed using WODL. Fluorescence indicates substrate degradation (bottom image). The top image shows the absence of fluorescence when the droplets contained only the substrate. (b) Microorganisms were cultured with fluorescent substrates in the WODL for 24 h. Pm and Ec indicate *P. mexicana* and *E. coli*, respectively. Fluorescence was observed in both cases. (c) Fluorescence intensity was measured using FADS. The fluorescence intensity values and standard deviations were calculated from approximately 3,000 droplets for each sample. FADS histograms are shown in Fig. S5. (d) FADS histogram of the analysis of droplets generated from a mixture of *E. coli* (lower fluorescent intensity) and *P. mexicana* (higher fluorescence intensity) (e) Results of WODL separation using FADS. The higher activity areas (with *P. mexicana* alone and *P. mexicana* and *E. coli* together) and lower activity areas (with *E. coli* alone) in panel d were separated based on fluorescence intensity. Scale bar represents 100 µm. NC, negative control; PC, positive control.

To verify the detectability of the endopeptidase activity produced by microorganisms in WODLs, we incubated purified trypsin, *P. mexicana*, and *E. coli* with a substrate within the WODLs. Proteolytic activity was assessed using the EnzChek Peptidase/Protease Assay Kit, which utilizes casein extensively labeled with the green-fluorescent BODIPY FL dye. In its intact form, the fluorescence of the dye is quenched due to the high density of labeling. Upon proteolytic cleavage by enzymes such as trypsin, labeled peptide fragments are released, resulting in a measurable increase in fluorescence intensity. Following a 24 h incubation period with 1 mg/mL trypsin and substrate, we observed an increase in green fluorescence, which can be attributed to substrate degradation. This phenomenon was not observed when only the substrate was encapsulated (Fig. 2a). We subsequently used *P. mexicana* and *E. coli* cultures to encapsulate the substrate and individual cells within droplets. The probability of cell inclusion was calculated from the Poisson distribution, with λ set at 0.314 (*P. mexicana*) and 0.213 (*E. coli*). An increase in green fluorescence was observed in the bacteria-containing droplets under a microscope after 24 h (Fig. 2b). FADS analysis of the fluorescence values showed an increase, predominantly in droplets encapsulated with trypsin and bacteria. *P. mexicana* displayed a higher fluorescence value than *E. coli* and even exceeded that of trypsin (Fig. 2c). This could be attributed to the diverse endopeptidase activities of *P. mexicana*. The endopeptidase produced by *P. mexicana* has substrate specificity similar to that of trypsin, which is primarily specific for basic amino acids. Additionally, the extracellular peptidase activity of *P. mexicana* demonstrated specificity for other amino acids (Fig. 6b), suggesting a cooperative degradation of peptide sequences that are resistant to cleavage by trypsin’s substrate specificity.

One of the significant advantages of a droplet-based microfluidic system for microorganism screening is its ability to compartmentalize. To demonstrate this, we premixed *E. coli* and *P. mexicana* before the formation of WODLs and subsequently isolated each bacterium-encapsulated WODL based on the endopeptidase activity exhibited by each bacterium. FADS was able to differentiate between empty droplets, those encapsulating *P. mexicana*, and those encapsulating *E. coli* (Fig. 2d). This analysis also indicated that the distribution of droplets containing each bacterium roughly followed a Poisson distribution with λ = 0.250 (for *P. mexicana*) and 0.173 (for *E. coli*) (Fig. S3). The intensity of the green fluorescence enabled us to distinguish between areas of higher and lower activity. Specifically, WODLs in the areas with higher fluorescence were expected to contain solely *P. mexicana* and those containing both *P. mexicana* and *E. coli*. Conversely, in the lower-fluorescence areas, where we anticipated finding droplets containing only *E. coli*, droplets were collected and re-cultured on agar medium (Fig. 2e). PCR analysis of the resulting colonies confirmed that isolation based on green fluorescence led to the exclusive isolation of *E. coli* (Fig. S4).

### Ultrahigh-throughput screening of microorganisms based on endopeptidase activity

**Fig 3:**
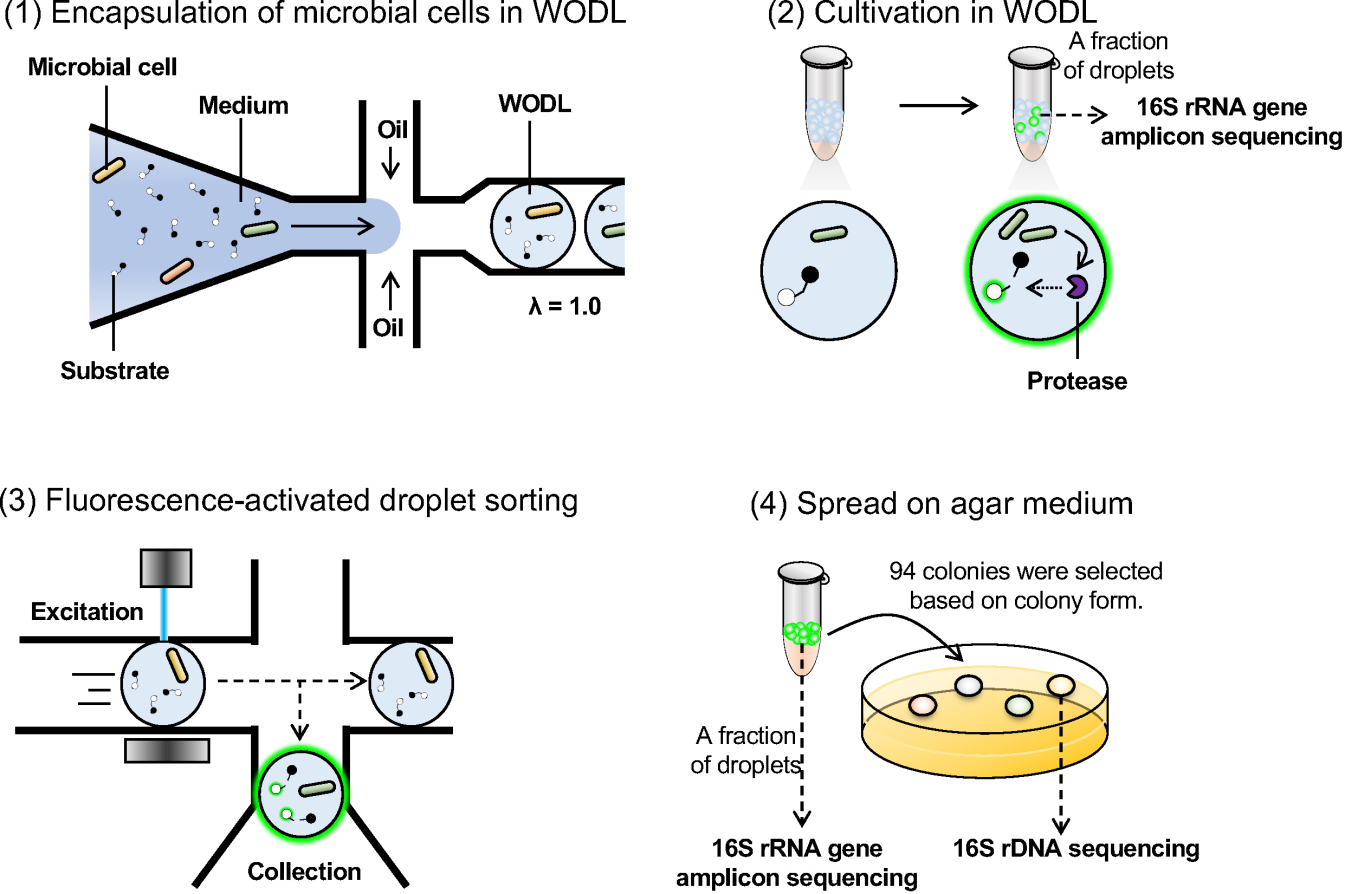
Schematic of the workflow for the isolation of environmental microorganisms based on endopeptidase activity (1). Perfluorocarbon oil, fluorinated surfactants, and casitone medium were used for encapsulation of microbial cells in WODLs (2). Droplets were incubated at 30°C for 3 days for cultivation in WODL (3). Droplets subjected to fluorescence activation were collected. The total number of droplets analyzed and droplets sorted was 1,002,443 and 10,356, respectively (4). The droplets were grown and analyzed further. Ninety-four colonies were randomly selected based on colony shape.

**Fig 4:**
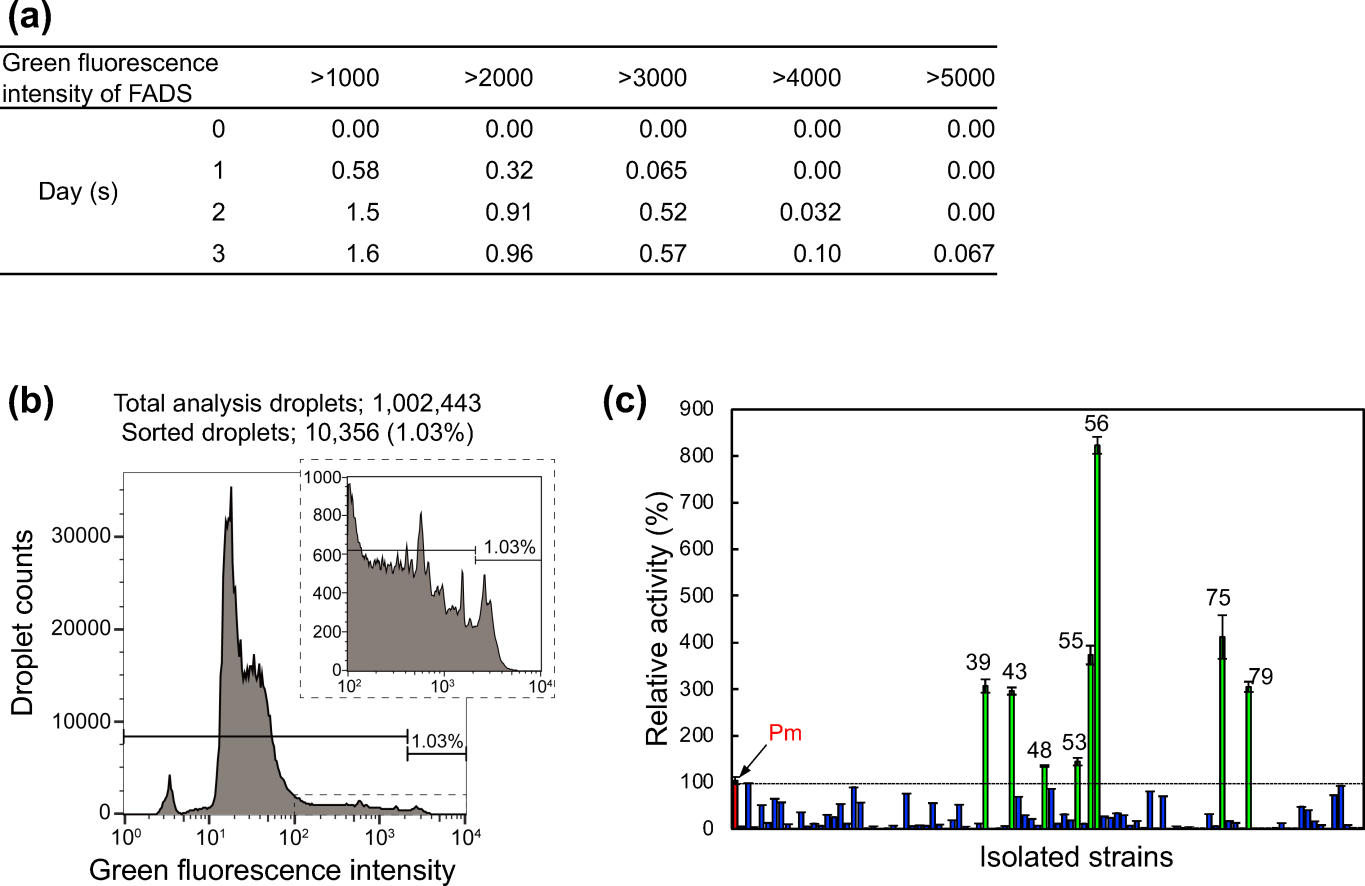
Droplet screening of environmental microorganisms based on endopeptidase activity. (a) Droplet ratio for each green fluorescence intensity by FADS. Each value (%) is the ratio obtained by dividing the number of droplets that show fluorescence intensity exceeding each green fluorescence intensity (1,000, 2,000, 3,000, 4,000, and 5,000) by the number of droplets analyzed. The green fluorescence intensity of approximately 3,000 droplets was measured at each culture time point using on-chip sorting, and the proportion of each fluorescence intensity was calculated. (b) FADS histogram of droplet screening for environmental microorganisms. FADS was performed after 3 days of droplet culture. The horizontal line in the histogram indicates the threshold range for sorting droplets. Approximately 1% of the droplets were isolated and spread on an agar medium. (c) Endopeptidase activity of the isolated microorganisms. Enzyme activity in the supernatant (intensity/min/mg protein) represents the relative activity when *P. mexicana* was set at 100% (red bar). *P. mexicana* and strain numbers 39, 43, 48, 53, 55, 56, 75, and 79 strains (green bars) were measured in three experiments. Error bars indicate standard deviation. For the other candidate strains (blue bars), the enzyme activity was determined using a single experiment.

Ultrahigh-throughput screening of the environmental microbes using droplets was conducted, as shown in Fig. 3. Microorganisms extracted from the soil were encapsulated in an emulsion using an EnzChek Peptidase/Protease Assay Kit substrate at a concentration of 10 µg/mL. The average encapsulation rate (*λ*) was set to 1.0. As cultivation time progressed, microbial growth and increased fluorescence intensity were observed in some droplets (Fig. S6). The proportion of droplets showing endopeptidase activity at each cultivation time was calculated (Fig. 4a). As the cultivation time increased, the proportion of droplets with higher fluorescence intensity also increased. This is due to the varying cultivation times required for substrate decomposition by microbial growth and enzymatic activity. Moreover, fluorescence intensity values over 4,000 were detected from the second day of cultivation, and values over 5,000 were detected from the third day, indicating significant differences in peptidase activity among the microorganisms.

On the third day of droplet cultivation, a histogram displaying the distribution of the green fluorescence intensity revealed a large peak in the range of 10–20 (Fig. 4b). This peak likely corresponds to empty droplets or those in which microbes did not grow, similar to the fluorescence distribution observed immediately after encapsulation (Fig. S7). In contrast, droplets where microbes grew, based on differences in endopeptidase activity, the range of fluorescence intensities was largely found to be over 20. To acquire more active microbes, droplets with the top 1% of high fluorescence intensity were sorted. In total, 1,002,443 droplets were analyzed, and approximately 1% (10,356 droplets) were sorted. The sorting process, including setting up of equipment and refilling and draining out the used sheath solution, took a total of 6 h. The encapsulation rate at *λ* = 1 was 63%, indicating that approximately 630,000 microorganisms were analyzed at the time of screening. It would have been impossible to analyze this many microbes within 6 h using conventional screening methods, which demonstrates the ultrahigh efficiency of droplet screening. This ability has been demonstrated in previous studies. For instance, Najah et al. isolated microbes in droplets based on cellobiohydrolase activity from soil, reporting 100,000 microorganisms screened in 20 min (17). Furthermore, droplet-based ultrahigh-throughput systems have been applied to the cell-free directed evolution of proteases (21) and the profiling of protease specificities (22), underscoring the power and versatility of microfluidics-based screening for proteolytic functions.

Microorganisms were collected from sorted droplets and isolated by spreading on agar medium. To identify microbes with higher activity, secondary screening was conducted based on cultivation using microplates and enzymatic activity measurements of the supernatant. Ninety-four strains were selected from the colonies isolated on the agar medium based on colony shape (Fig. S8). At this time, *P. mexicana* (which had relatively high endopeptidase activity) and *E. coli* (which had relatively low activity) were cultivated simultaneously. After 5 days, the culture broth was centrifuged, the supernatant was collected, and the protein quantity and endopeptidase activity were measured. Activity was calculated as the fluorescence intensity increase per minute per 1 mg of supernatant protein and displayed as relative activity when *P. mexicana* activity was taken as 100% (Fig. 4c). Strains 39, 43, 48, 53, 55, 56, 75, and 79 showed relative activities exceeding 100%. To identify these eight strains, the 16S rRNA gene region was amplified and sequenced within a range of 27–1,500 bp. The isolated strains were classified into four species: *Stenotrophomonas maltophilia* (No. 39, No. 43, No. 55, and No. 79), *Dyella koreensis* (No. 48), *Lysobacter soli* (No. 53), and *Lysobacter enzymogenes* (No. 56 and No. 75), summarized in Table 1.

**TABLE 1:** List of isolated strains possessing high endopeptidase activity

No.	Strain	16S rRNA gene identity (%)	Accession no.
39	*Stenotrophomonas maltophilia strain* ATCC13637	99.21	NR_112030.1
43	*Stenotrophomonas maltophilia strain* ATCC13637	99.21	NR_112030.1
48	*Dyella koreensis* strain NBRC100831	100.00	NR_113947.1
53	*Lysobacter soli* strain DCY21	99.21	NR_116074.1
55	*Stenotrophomonas maltophilia* strain ATCC13637	99.28	NR_112030.1
56	*Lysobacter enzymogenes* strain 495	99.93	NR_036925.1
75	*Lysobacter enzymogenes* strain 495	99.93	NR_036925.1
79	*Stenotrophomonas maltophilia* strain ATCC13637	99.28	NR_112030.1

**Fig 5:**
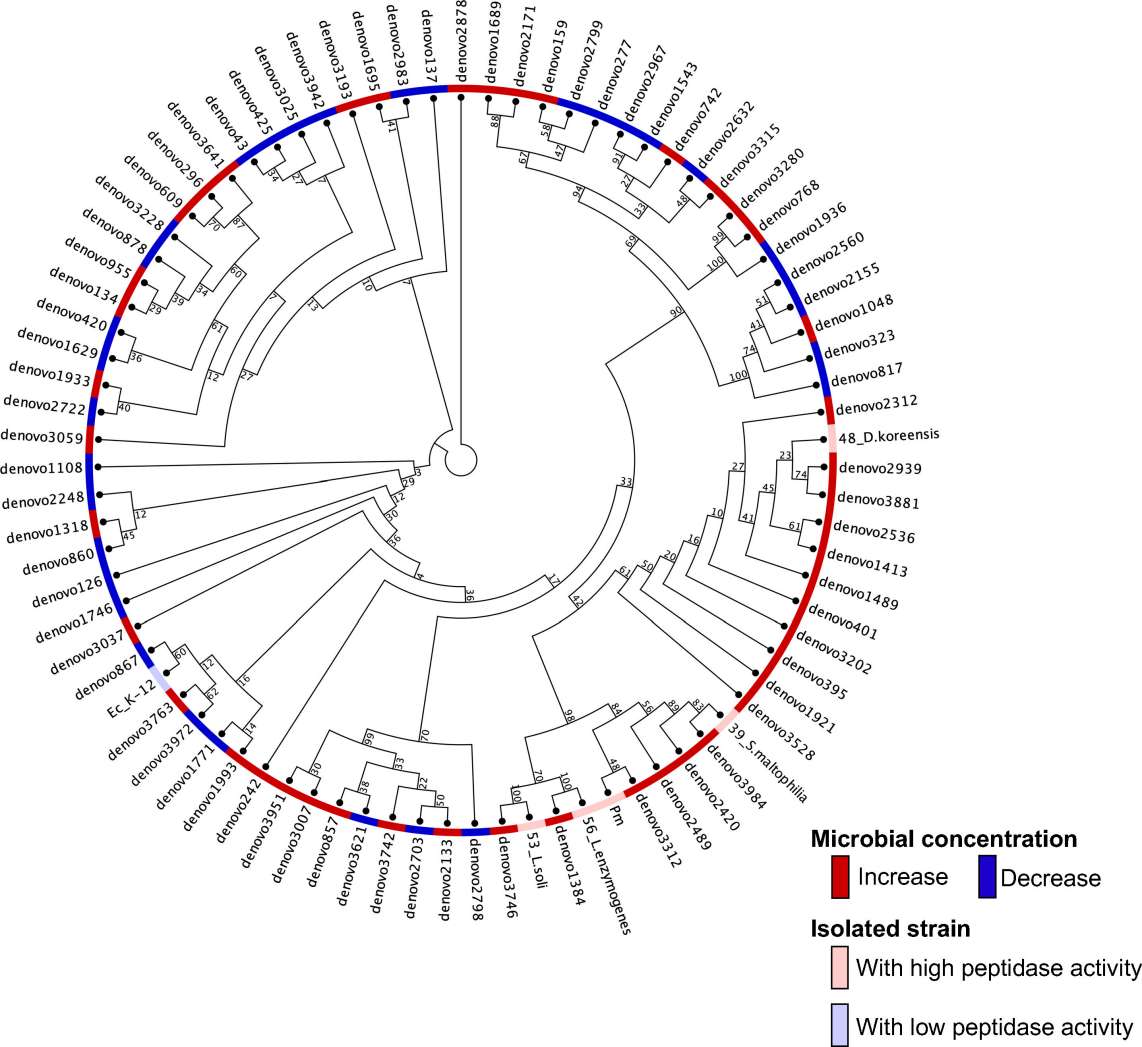
Phylogenetic analysis in post-droplet cultivation and post-sorting samples. The nucleotide sequences of each OTU were aligned using the CLC Genomics Workbench, and a phylogenetic tree was constructed using the neighbor-joining method. During this process, the bootstrap test was set to 1,000 replicates. Microorganisms classified into each OTU that showed an increased abundance ratio were labeled in red, whereas those with a decreased ratio were labeled in blue.

To compare the changes in microbial composition before and after sorting for confirming whether sorting enriched the microorganisms with high peptidase activity, genomic DNA was extracted from samples post-droplet cultivation and post-sorting, and 16S rDNA amplicon sequence analysis was performed using MiSeq (Fig. S9). The enrichment rate of each operational taxonomic unit (OTU) by FADS was calculated by dividing the amount present in the post-sorting sample by the amount present in the post-droplet cultivation sample. Based on this, OTUs sorted for high-peptidase activity should have an enrichment ratio greater than 1, and conversely, OTUs with no or low peptidase activity should be less than 1. After calculating the enrichment ratio for each detected OTU, we found that there were 45 OTUs with an enrichment ratio exceeding 1 and 35 OTUs with an enrichment ratio of less than 1. To examine the phylogenetic distribution of these functionally differentiated taxa, we constructed a neighbor-joining phylogenetic tree of the identified OTUs (Fig. 5). The outer ring of the tree indicates enrichment status (red for increased and blue for decreased), while strains isolated and confirmed to have high peptidase activity are highlighted in pink (isolated strains) and light blue (confirmed lower activity). Notably, OTUs closely related to the isolated strains (e.g., *S. maltophilia*, *D. koreensis*, *L. soli*, and *L. enzymogenes*) were frequently enriched, suggesting that high endopeptidase activity may be phylogenetically conserved, a phenomenon reported for other microbial enzymatic traits as well (23). The observed concentration rate of specific microbial taxa obtained through droplet cultivation and function-based droplet sorting (e.g., based on peptidase activity) suggests that the enrichment process reflects both phylogeny-dependent cultivation efficiency and functional screening biases. Certain phylogenetic groups may be inherently more amenable to growth under droplet conditions and/or more likely to exhibit the targeted enzymatic activity. Therefore, the resulting phylogenetic distribution is shaped by a combination of cultivation and screening factors. Given that droplet compartmentalization has been shown to reduce cultivation bias compared to conventional bulk culture (17), we propose that the droplet-based microfluidics method is an efficient alternative for function-based environmental microbial screening.

### Identification of isolated microorganisms and peptidase

**Fig 6:**
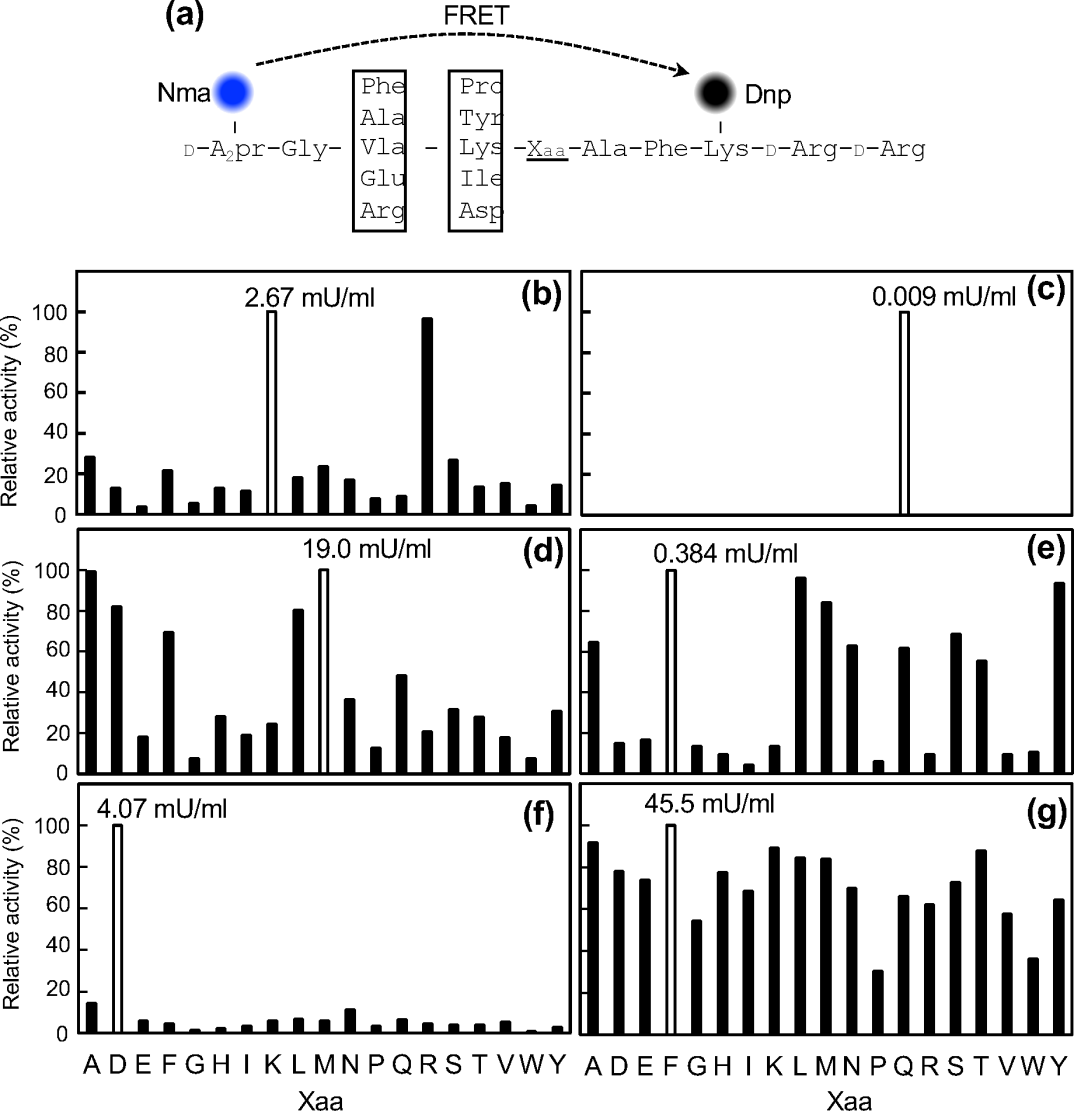
Substrate specificity analysis of endopeptidases produced by each microorganism. (a) Schematic representation of FRETS-25Xaa substrates used. Substrate specificity of *P. mexicana* (b), *E. coli* (c), *S. maltophilia* (d), *D. koreensis* (e), *L. soli* (f), and *L. enzymogenes* (g). Horizontal axis represents the type of amino acid for Xaa, and the vertical axis represents relative activity (IU/mL) when the highest activity among the 19 amino acids was set to 100%. Numbers in the graph indicate the highest activity values (white columns).

Using droplet cultivation and microtiter plate cultivation for screening, we selected four strains with higher endopeptidase activity than that of *P. mexicana*. As the activity measured in the droplet-based assay reflects the cumulative proteolytic activity, it remains unclear whether the observed activity is attributable to a single dominant enzyme or multiple co-expressed proteases. Thus, the following enzyme characterizations aim to clarify potential differences in substrate specificity, which may reflect the diversity of peptidases present in each strain. Here, by examining whether each microorganism can cleave specific peptide chains or whether they correspond to a broad range of sequences, we characterized the substrate specificity of the enzymes they produce. In addition to the four isolated microbial species, we cultured the model microbes *P. mexicana* and *E. coli* in microplates and measured their activities in the supernatants. We used the FRETS-25Xaa series (from the Peptide Research Institute) as substrates, where the P1 site of the peptide is composed of each of the 19 types, excluding cysteine, to analyze substrate specificity (Fig. 6a).

The endopeptidase produced by *P. mexicana* showed high activity against the basic amino acids Lys and Arg (Fig. 6b). *S. maltophilia* and *D. koreensis* displayed high activity primarily toward hydrophobic amino acids (Fig. 6d and e). *E. coli* and *L. soli* exhibited specific activity against Gln and Asp (Fig. 6c and f). *L. enzymogenes* demonstrated broad activity, recognizing a wide range of amino acids (Fig. 6g). Thus, even among the four strains isolated with high endopeptidase activity, their substrate specificity varied significantly.

The endopeptidase secreted by *S. maltophilia* exhibits high specificity for hydrophobic amino acids such as Ala and Met. This is believed to be due to the involvement of Family S8 serine peptidases, such as StmPr1, StmPr2, and StmPr3, that are present in this species for recognizing and cleaving hydrophobic amino acids (24, 25). *D. koreensis*, which also showed specificity for hydrophobic amino acids, is known as a β-glucosidase-producing bacterium, but not much information is available regarding its peptidases in literature (26) as well as in the peptidase database MEROPS, suggesting it might be a novel high-peptidase-producing microorganism. The endopeptidase produced by *L. enzymogenes* demonstrates specificity toward a broad range of amino acids, suggesting that multiple enzymes might be working in tandem in this case. Indeed, it is known that the *L. enzymogenes* M497-1 strain possesses over 117 peptidase genes (27), of which 80 peptidase genes are present within the gene cluster, and a further 16 peptidase genes showed higher expression levels than the 16S rRNA transcript, which is commonly used as a reference due to its stable and abundant expression. Many other *Lysobacter* species also possess numerous peptidase genes, suggesting that the strain isolated in this study might harbor a diverse range of peptidases. In contrast, the substrate specificity of *L. soli* is specific to Asp, a result that differs from the broad specificity of *L. enzymogenes*. Like other *Lysobacter* species, it might also possess numerous peptidase genes; however, under the current cultivation conditions, our results suggested that a single type of endopeptidase might be predominantly expressed. Additionally, *L. soli* was reported as a microorganism isolated from a ginseng field in China, but there are hardly any reports describing its enzyme production (28). Therefore, in this study, we isolated, purified, and characterized the Asp-specific endopeptidase derived from *L. soli*.

### Characterization of the Asp-specific peptidase from *L. soli*

**Fig 7:**
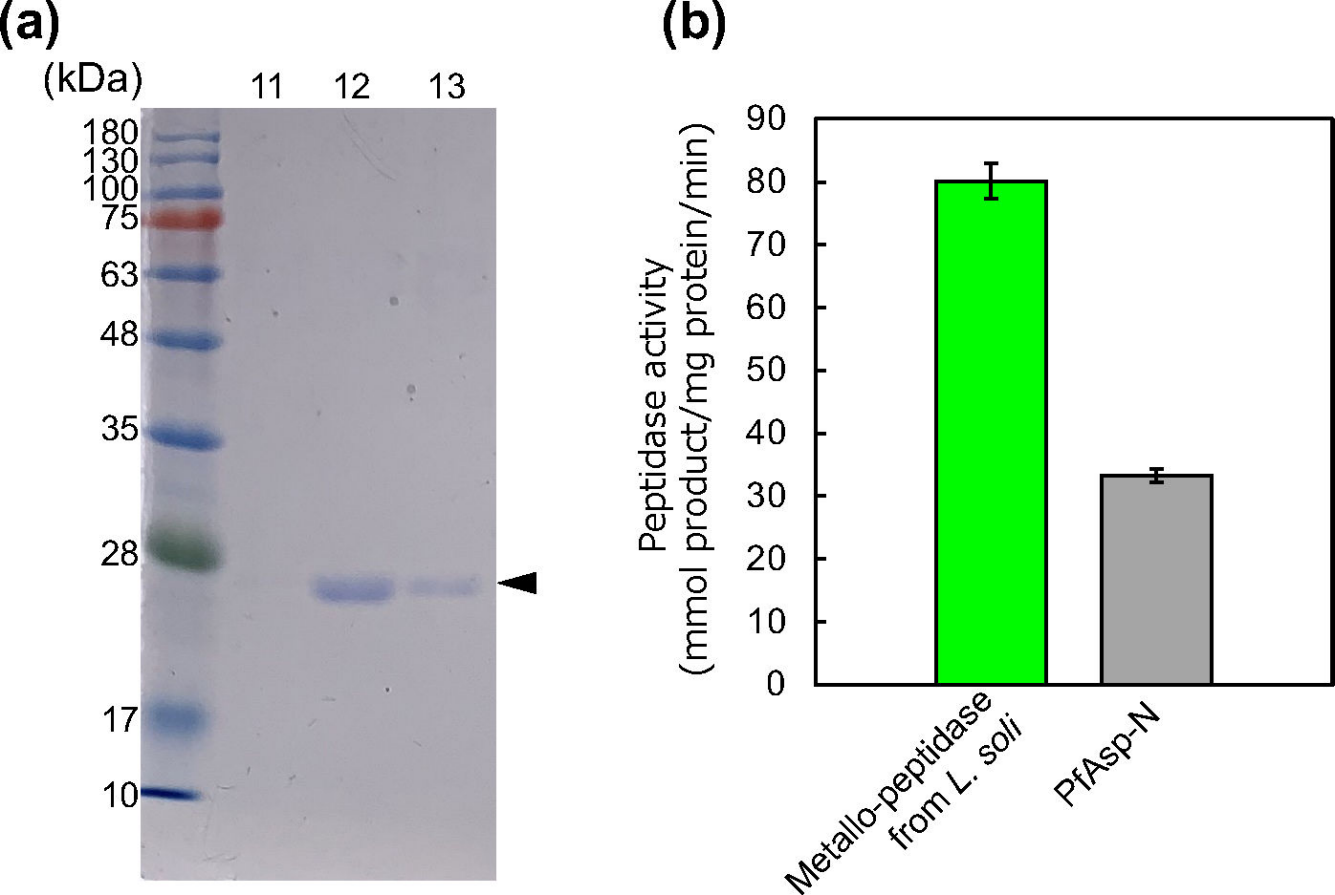
Purification and characterization of a metallo-peptidase from *L. soli*. (a) Coomassie-stained 10% SDS–PAGE analysis of purified metallo-peptidase from *L. soli*. Molecular-weight markers of different masses (kDa) are labeled. Lane numbers represent the fraction numbers of gel filtration chromatography. (b) Determination of specific activity for FRETS-25-Asp substrate.

To characterize the Asp-specific endopeptidase derived from *L. soli*, the native enzyme was first purified. The supernatant from *L. soli* cultured for two days was concentrated and purified by anion-exchange chromatography and gel filtration chromatography (Fig. 7a; Fig. S10). Upon analyzing the N-terminal amino acid sequence of the purified enzyme, a gene annotated as the M12 family metallopeptidase was identified in the genome. A related enzyme resulting from Basic Local Alignment Search Tool (BLAST) in the Swiss-Prot database, peptidyl-Asp metallo-endopeptidase derived from *S. maltophilia* (SmAsp-N) and *Pseudomonas fragi* (PfAsp-N), was identified. In particular, PfAsp-N is an industrial digestion enzyme, used for peptide fingerprinting (29, 30). In this study, we compared its relative activity with that of the commercially available PfAsp-N. Surprisingly, the Asp-specific endopeptidase from *L. soli* exhibited 2.4 times more activity compared to that of PfAsp-N (Fig. 7b). Although the total length of Asp-specific endopeptidase from *L. soli* in the database exceeded 500 amino acids, the results obtained from N-terminal analysis indicated that this endopeptidase is a short fragment of the enzyme (Fig. S11). PfAsp-N is also a short peptidase with a total length of 144 amino acids. Moreover, Asp-specific endopeptidase from *L. soli* possesses a P-domain involved in the function of the propeptide in eukaryotes (31). From these facts, it can be inferred that some processing is required for the maturation of Asp-N. We attempted to verify the presence or absence of activity of the full-length Asp-specific endopeptidase through heterologous host expression utilizing *E. coli*; however, verification was not possible due to the protein being expressed as an insoluble fraction. Although this point should be clarified in greater detail, such an explanation is beyond the scope of this paper. In conclusion, in this paper we report the successful isolation of a related peptidase with higher activity than the existing known industrial enzymes through this large-scale screening using droplets.

### Conclusion

The present study emphasizes the importance and the unique potential of microbial screening using the droplet-based microfluidics systems in uncovering novel enzymes from environmental microbes. In particular, the Asp-specific protease obtained in this study, when screened based on endopeptidase activity, was found to be 2.4 times more active than the related commercially available enzymes. Even though *in silico* analysis can predict the activity status, it cannot predict the extent of enzyme activity. Our results indicate the ability of this function-based screening to provide more critical information that is crucial for acquiring useful enzymes. The successful isolation of industrially relevant endopeptidases demonstrates the efficacy of this method, setting the stage for its broader application in industrial microbiology.

## MATERIALS AND METHODS

### Materials, bacterial strains, medium, and enzymes

Enzchek Peptidase/Protease Assay Kit (Thermo Fisher Scientific, MA, USA) was used as endopeptidase substrate (currently not available, but EnzChek Protease Assay Kits, which can be purchased from Thermo Fisher Scientific, is an alternative). Two types of bacteria were used in the present study. *E. coli* DH5α (Takara Bio Inc., Japan), derived from the K12 strain, was used as a low-peptidase-producing bacterium. *P. mexicana* WO24, which was isolated by Ogasawara et al. (32), was used as a peptidase-producing bacterium. Soil sample was collected from the Nagaoka University of Technology in Japan (37°25′N, 138°46′E). Casitone medium: 1% (wt/vol) Bacto Casitone (Becton, Dickinson and Company, NJ, USA), 0.2% (wt/vol) Bacto Yeast extract (Becton, Dickinson and Company), and 4 mM MgSO_4_ (Nacalai Tesque, Inc., Japan). The casitone agar medium consisted of casitone medium and 1.5% (wt/vol) agar (Nacalai Tesque, Inc.). Lysogeny broth (LB) medium: 1% (wt/vol) Bacto Tryptone (Thermo Fisher Scientific), 0.5% (wt/vol) Bacto Yeast extract (Becton, Dickinson and Company), and 1% (wt/vol) NaCl (Nacalai Tesque, Inc.). Trypsin and Endoproteinase Asp-N were purchased from Merck (Germany) and Takara Bio, Inc. (Japan), respectively.

### WODLs generation, sorting, and imaging

WODLs with a diameter of approximately 120 µm were produced using an On-chip Droplet Generator (On-chip Biotechnologies, Japan). The oil phase was HFE-7500 Novec Engineered fluid (HFE-7500), which contained 2% (wt/wt) Pico-Surf 1 (Sphere Fluidics, UK) or 2% (wt/wt) 008-FluoroSurfactant (RAN Biotechnologies, MA, USA) as a surfactant. The perfluorocarbon oil used for droplet generation is highly permeable to gases, including oxygen, and enables aerobic cultivation in emulsified systems (33). FADS was performed using On-chip Sort (On-chip Biotechnologies) at a rate of about 100 droplets/s. 0.1% (wt/wt) Pico-Surf 1 in HFE-7500 or 0.1% (wt/wt) 008-FluoroSurfactant in HFE-7500 was used as the sheath solution. Micrographs were obtained using a laser-scanning confocal microscope system A1 (Nikon, Japan) operated by the NIS-Elements software (Nikon). The WODLs were placed into a μ-Slide VI flat microscopy chamber (IBIDI, Germany) prefilled with 0.1% (wt/wt) Pico-Surf in HFE-7500 or 0.1% (wt/wt) 008-FluoroSurfactant in HFE-7500. Fluorescence intensity analysis was performed with ImageJ software (34).

### Turbidity measurement in WODL

Methods for the calculation of microbial occupancy are available in the literature (20). Microscopic images of droplets at each time point were imported into ImageJ and binarized to determine the edges of microbial cells in a WODL. The area excluding the microbial cells was extracted and measured (*A*_ex_). The measured area (*A*_ex_) and the droplet area (*A*_droplet_) were used to determine the occupancy (*A*_occup_) calculated as *A*_occup_(%) = (*A*_droplet_ – *A*_ex_)/*A*_droplet_ × 100. A calibration curve of OD_600_ and occupancy was created by sealing the droplet with a culture medium of known OD_600_ and determining occupancy (Fig. S1). The OD_600_ of the bulk culture was measured at each time point using a spectrophotometer (UVmini-1240; Shimadzu). Bulk cultures were performed under aerobic conditions at 30 °C with shaking at 180  rpm. The maximum growth rate and lag time were determined by fitting the experimental data to a growth curve using the ComBase database (www.combase.cc).

### Enzymatic reactions in the WODLs

A 10 mM Tris-HCl solution (pH 7.8) was used as the reaction buffer. Trypsin (1 mg/mL) was used to hydrolyze the Enzchek Peptidase/Protease Assay Kit at 10 µg/mL. This assay employs a casein substrate extensively labeled with the pH-insensitive, green-fluorescent BODIPY FL dye. In its intact form, the fluorescence of the dye is quenched due to close proximity labeling. Upon proteolytic cleavage by enzymes such as trypsin, the labeled peptide fragments are released, resulting in a significant increase in fluorescence intensity. These reaction mixtures were encapsulated in WODLs using an on-chip droplet generator. Enzymatic reactions were performed at 30°C.

### WODLs cultivation of *E. coli* and *P. mexicana*

*E. coli* and *P. mexicana* were precultured in casitone medium. The bacterial culture medium was centrifuged at 6,000 × *g*, and the pellet was washed with 0.9% (wt/vol) NaCl solution before being suspended in casitone medium. Stochastic encapsulation of microorganisms followed a Poisson distribution as per the previously investigated relationship between OD_600_ and colony forming unit (Fig. S3) (35). WODLs containing microorganism cells and substrate (Enzchek Peptidase/Protease Assay Kit) were statically cultivated at 30°C for 1 day.

### Colony-direct PCR

Colony-direct PCR was performed to identify *E. coli* and *P. mexicana* and sorted based on peptidase activity. Primers were designed specifically to amplify the V3–V4 region of *E. coli* (Fig. S4). PCR was performed using Quick Taq HS DyeMix (Toyobo Co., Ltd., Osaka, Japan) as the DNA polymerase. The template consisted of 12 randomly selected colonies. PCR conditions were set as follows: 1 cycle of 94°C for 2 min, 30 cycles of 94°C for 30 s, 55°C for 30 s, and 68°C for 30 s.

### Screening and isolation of microorganisms from the environment

For the soil sample, 5 g of soil was suspended in 35 mL of 0.9% NaCl, and the suspension was vortexed for 3 min. After centrifuging at 1,000 × *g* at 4°C for 5 min, 15 mL supernatant was collected. The supernatant was filtered through a membrane with a pore size of 50 µm (AS ONE Corporation, Japan) to remove the soil particles. The filtrate was centrifuged at 6,000 × *g* at 4°C for 5 min, and the pellet was washed with 30 mL of 0.9% NaCl. After centrifugation at 6,000 × *g* at 4°C for 5 min, the pellet was suspended in 4 mL casitone medium. Cells were stained using 1 µM SYTO9 (Thermo Fisher Scientific) and 1 µM propidium iodide (Thermo Fisher Scientific), and the stained cells were counted using a hemocytometer. Stochastic encapsulation of microorganisms was used to calculate the Poisson distribution using the number of cells. WODLs containing microorganism cells and substrate (Enzchek Peptidase/Protease Assay Kit) were statically cultivated at 30°C for 3 days. Approximately one million WODLs encapsulating soil samples in the top 1% of the fluorescence histograms were sorted. The casitone medium (100 µL) and surfactant-free HFE-7500 were added to the sorted WODLs to break the droplets. The aqueous phase was placed in a new tube and diluted 1,000-fold with the casitone medium. The diluent was spread on casitone agar medium. Bacterial colonies were isolated, cultured in casitone medium, and stored at –80°C.

### Measurement of proteolytic activity of isolated bacteria

The amount of protein and enzyme activity in the supernatant of each microorganism was measured after 5 days of incubation at 25°C in 1 mL of casitone medium. The enzymatic reaction was performed in a reaction buffer containing 10 mM Tris-HCl pH 7.8 at 30°C for 20 min. 50 µL of substrate solution and 50 µL of supernatant from the isolated bacterial culture were mixed to initiate the enzymatic reaction. For the endopeptidase activity measurement, the Enzchek Peptidase/Protease Assay Kit was used as substrate at 10 µg/mL (final concentration), and the fluorescence intensity of this substrate was measured with excitation at 490 nm and emission at 520 nm using an Infinite 200 PRO microplate reader (Tecan, Switzerland). To determine substrate P1 specificity, FRETS-25Xaa (Peptide Institute, Inc., Japan) was used as the substrate at a final concentration of 25 µM. This substrate consists of N-methylanthranilic acid (Nma) as the donor fluorophore and 2,4-dinitrophenyl (DNP) as the acceptor/quencher. In the intact peptide, fluorescence from Nma is efficiently quenched via fluorescence resonance energy transfer (FRET) to DNP (Fig. 6a). The fluorescence intensity of this substrate was measured with excitation at 340 nm and emission at 440 nm using an Infinite 200 PRO microplate reader. To quantify enzymatic activity, a calibration curve was constructed using FRETS-25-STD1 and FRETS-25-STD2 reference compounds (Peptide Institute, Inc.), which contain pre-cleaved Nma. The standard solutions ranged from 16 pmol to 1 nmol of Nma equivalent. The protein concentration in the supernatant of the isolated bacterial culture was determined by the Bradford assay (Quick Start Bradford 1×, Bio-Rad Laboratories), and a linear calibration curve was obtained using bovine gamma globulin with concentrations ranging from 0 to 0.25 mg/mL. Standard deviations were calculated from three independent experiments.

### Sequencing analysis

For metagenomic analysis, genomic DNA was extracted from the WODL sample as described in the literature (19). In simple terms, the WODLs were broken using Pico-Break (Sphere Fluidics Limited, UK), and the aqueous phase was collected and centrifuged. The pellet containing the bacteria was dissolved in lysis buffer containing lysozyme. Genomic DNA was purified using the standard DNA purification method. The methods of preparation of 16S rDNA library, sequencing, and data analysis followed the ones reported in the literature (36). A forward universal bacterial primer 515F (5′-GTGCCAGCMGCCGCGGTAA-3′) and a reverse universal primer 806R (5′-GGACTACHVGGGTWTCTAAT-3′) were used to amplify the bacterial 16S rRNA genes. PCR was performed using KOD FX Neo DNA polymerase (Toyobo Co., Ltd.). The conditions were set as follows: 1 cycle at 94°C for 2 min, 40 cycles at 98°C for 10 s, and 68°C for 30 s. PCR products were purified using a MinElute PCR Purification Kit (Qiagen, Germany) (Fig. S9), and the PCR product concentrations were measured using a Qubit 2.0 Fluorometer (Thermo Fisher Scientific). 16S rRNA gene sequencing was performed as described in the literature (37). DNA was sequenced using a MiSeq Reagent Kit v2 and the MiSeq System (Illumina Inc., CA, USA). Metagenomic sequencing data were analyzed using the Quantitative Insights into Microbial Ecology software (version 1.9.1) (38). OTUs were selected at 97% identity with UCLUST. Taxonomic classification was performed using the BLAST based on the Greengenes database ver. 13_8. The phylogenetic tree was constructed by MUSCLE multiple sequence alignment and the neighbor-joining method on the CLC Genomics Workbench. The percentage of replicate trees in which the associated taxa clustered together in the bootstrap test (1,000 replicates) is shown next to the branches. The metagenomic sequencing data in this study were obtained from the DNA Data Bank of Japan database under accession number PRJDB19635.

For isolated microorganisms, between 27 and 1,500 bp of 16S rRNA genes were amplified using the forward primer 27F (5′-AGAGTTTGATCATGGCTCAG-3′) and reverse primer 1,500R (5′-TACCTTGTTACGACTT-3′), and each colony was used as a template. PCR was performed using KOD FX Neo DNA polymerase (Toyobo Co., Ltd.). The conditions were set as follows: 1 cycle at 94°C for 2 min, 35 cycles at 98°C for 10 s, 55°C for 30 s, and 68°C for 2 min. PCR products were sequenced using the Sanger method, carried out by Eurofins Genomics (Japan). BLAST in the NCBI database (http://blast.ncbi.nlm.nih.gov/Blast.cgi) was used for searching the bacterial species corresponding to the 16S rDNA.

### Purification and characterization of Asp-specific peptidase

In our preliminary study, when cultivating *L. soli* in casitone medium, peptidase activity in the supernatant peaked on the second day. Therefore, for purification, we used the supernatant from the 2-day cultivation. After centrifuging and filtering approximately 3,600 mL (8.3 µg/mL total protein concentration) of the culture medium from the second day of cultivation to remove impurities, the supernatant was concentrated up to approximately 4 mL (443.8 µg/mL total protein concentration) using a 3 kDa ultrafiltration membrane while exchanging with 50 mM Tris-HCl buffer (pH 7.8). The ultrafiltrate was purified by anion-exchange chromatography using a HiTrap DEAE FF column (Cytiva, Marlborough, MA, USA). Proteins were eluted using a linear concentration gradient of 0–0.5 M NaCl. We collected fractions showing Asp-specific protease activity to a volume totaling 3.6 mL and concentrated them to 200 µL using ultrafiltration. For the activity measurement, the FRETS-25 aa series FRETS-25-Asp (Peptide Institute Inc.) was used as the substrate (Fig. 6a). The concentrate was further purified by gel filtration chromatography using Superdex 75 10/300 Gl column (Cytiva). Based on the results of Coomassie-stained 12% SDS–PAGE analysis, we confirmed that the fraction showing Asp-specific protease activity contained a single protein band of approximately 25 kDa and used this sample for characterization. The N-terminal amino acid sequence analysis of the purified enzyme was entrusted to Hokkaido System Science Co., Ltd. N-terminal amino acid sequencing identified a gene annotated to the M12 family metallopeptidase (NCBI Reference Sequence: WP_198173817.1). Alignment analysis of Asp-N sequences was performed using MUSCLE multiple sequence alignment on the CLC Genomics Workbench. Asp-Ns from *S. maltophilia* (SmAsp-N) and *P. fragi* (PfAsp-N) were obtained by BLAST in the Swiss-Prot database using the amino acid sequence of metallopeptidase from *L. soli* as the query sequence. The accession numbers of UniProtKB for SmAsp-N and PfAsp-N are B2FQP3 and Q9R4J4, respectively. InterPro release 97.0 was used for secondary structure prediction (39).

### Graphical programs

Histograms and plots of FADS were generated using FlowJo software version 10.7.1 (Becton, Dickinson and Company). Phylogenetic tree and protein alignments were performed using CLC Genomics Workbench version 23.0.4.

## SUPPLEMENTAL MATERIAL

Supplemental figures aem.00109-25-s0001.pdf Figures S1 to S11.

ASM does not own the copyrights to Supplemental Material that may be linked to, or accessed through, an article. The authors have granted ASM a non-exclusive, world-wide license to publish the Supplemental Material files. Please contact the corresponding author directly for reuse.
