# Supplementary material for: Ultrahigh-throughput screening of environmental bacteria for proteolytic activity using droplet-based microfluidics
Source: Appl Environ Microbiol. 2025 Jun 13;91(7):e00109-25. doi: 10.1128/aem.00109-25 (PMC12285256; doi:10.1128/aem.00109-25)
Supplement: Supplemental figures — Figures S1 to S11. [file aem.00109-25-s0001.pdf]

Supplemental Material

Ultrahigh-throughput screening of environmental bacteria for proteolytic activity using droplet-based microfluidics

Akihiro Nakamura,[1] Yoshiyuki Suzuki,[1] Nobuyuki Homma,[2] Yosuke Shida,[3] Rikako Sato,[4] Hiroaki Takaku,[4] and Wataru Ogasawara.[1,3]*

[1]Department of Science of Technology Innovation, Nagaoka University of Technology, 1603-1 Kamitomioka, Nagaoka-shi, Niigata 940-2188, Japan.

[2]On-chip Biotechnologies Co., Ltd., 2-16-17, Naka-cho, Koganei-shi, Tokyo 184-0012, Japan.

[3]Department of Materials Science and Bioengineering, Nagaoka University of Technology, 1603-1 Kamitomioka, Nagaoka-shi, Niigata 940-2188, Japan.

[4]Department of Applied Life Sciences, Niigata University of Pharmacy and Medical and Life Sciences, Higashijima 265-1, Akiha-ku, Niigata 956-8603, Japan.

*Correspondence and requests for materials should be addressed to
W.O. (owataru@vos.nagaokaut.ac.jp)

Supplemental material includes:

Figures S1-S11;

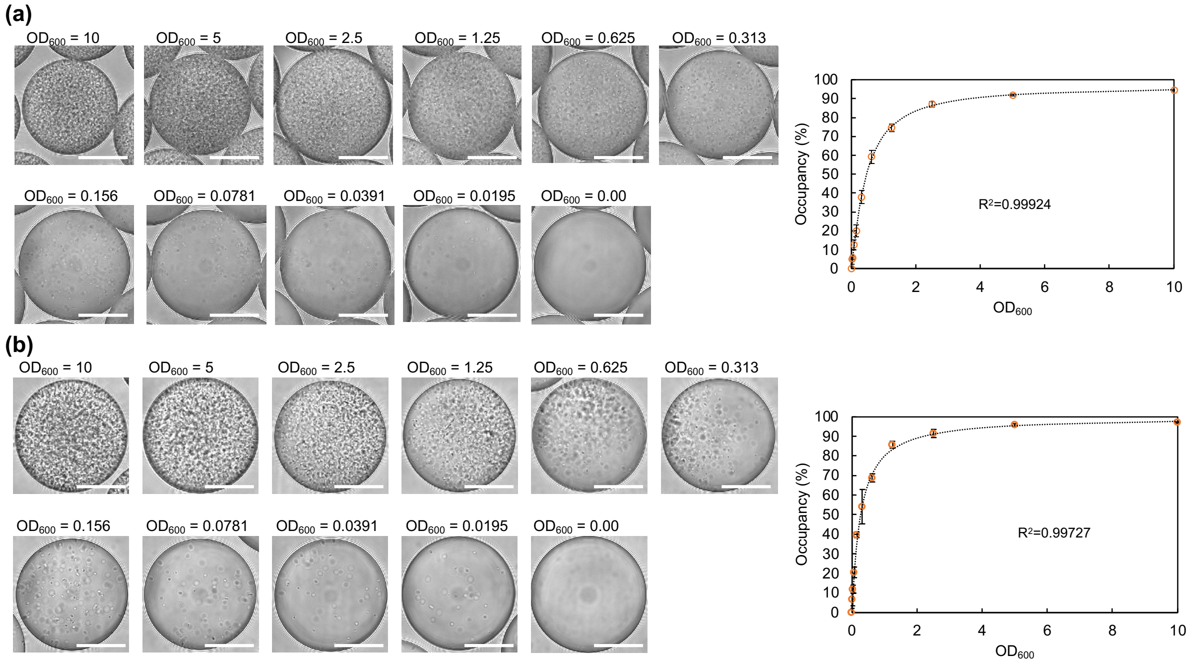

Figure S1 | Calibration curves of OD_{600} and droplet occupancy.

Microscopic images of droplets of *P. mexicana* WO24 (a) and *E. coli* (b). Calibration curves were created by regressing on a four-coefficient logistic curve using actual measurements. Standard deviations were obtained from 5 droplets. The scale bar represents 100 µm.

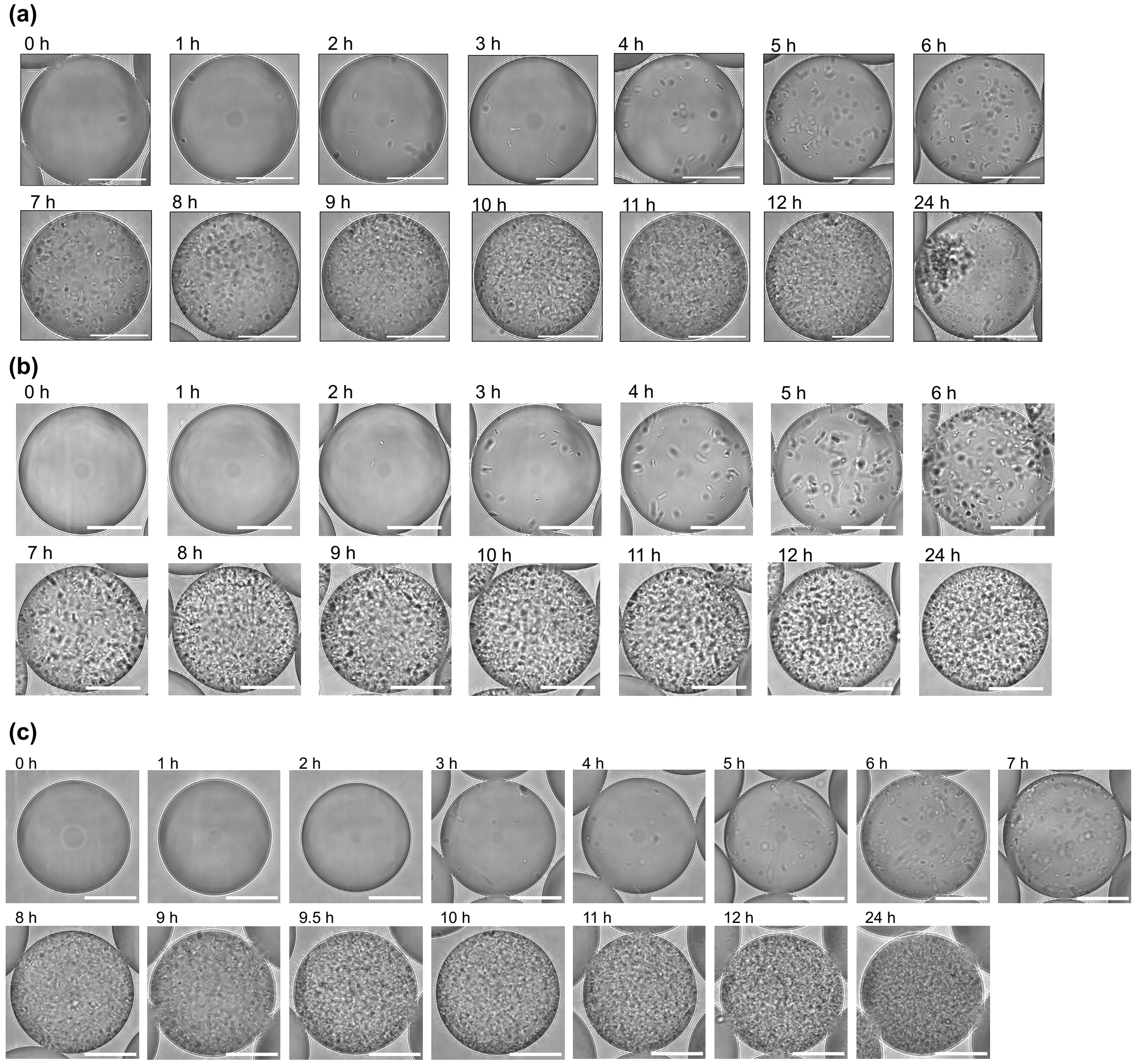

Figure S2 | Microscopic observation of droplet cultivation at each incubation time.

(a) *E. coli* DH5α was cultivated in casitone medium. (b) *E. coli* DH5α was cultivated in LB medium. (c) *P. mexicana* was cultivated in casitone medium. Droplets were not photographed continuously; instead, they were intermittently sampled and imaged. Droplets containing microorganisms were randomly selected from the imaged set and are presented here.

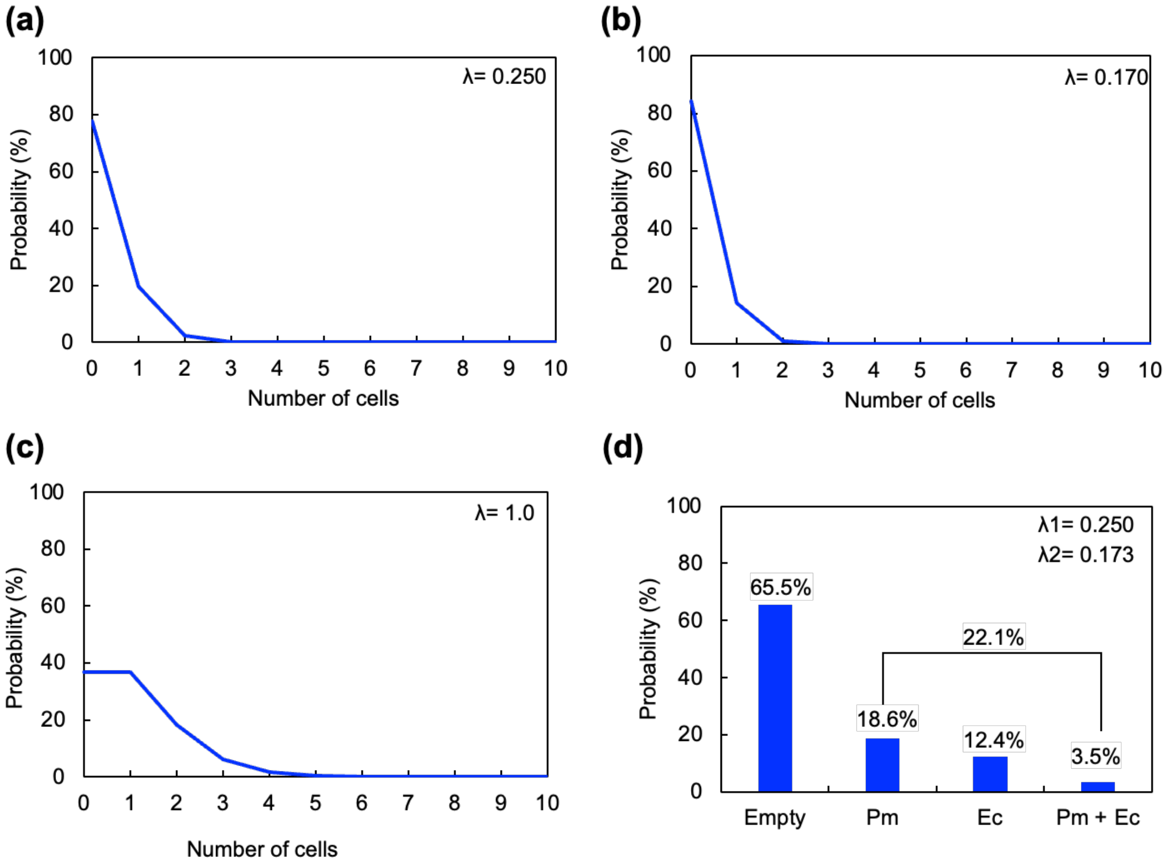

Figure S3 | Poisson distribution of encapsulation of microorganisms.

The probability $P(X = x)$ of finding x entities (bacterial cells) was calculated by the equation $P(X = x) = e^{-\lambda}(\lambda^x/x!)$, where *e* is Napier's constant and λ is the number of bacterial cells in each droplet volume. λ set at 0.250 for *P. mexicana* WO24 (a) and at 0.170 for *E. coli* (b), related to Figure 1 and 2a-c. For the environmental microorganisms (c), λ set at 1.0 in the screening experiment related to Figure 4. (d) The probability of co-encapsulation $P(X_1 = x_1, X_2 = x_2)$ of x_1 cells (*P. mexicana* WO24; Pm) and x_2 cells (*E. coli*; Ec) in a single droplet was calculated by the equation $P(X_1 = x_1, X_2 = x_2) = [e^{-\lambda 1}(\lambda_1^{x1}/x_1!)] \times [e^{-\lambda 2}(\lambda_2^{x2}/x_2!)]$. Here, λ was set at 0.250 for *P. mexicana* WO24 and at 0.173 for *E. coli* in the experiment related to Figure 2d-e. 'Empty' refers to an empty droplet with no microorganisms enclosed. 'Pm' is a droplet in which only *P. mexicana* WO24 was enclosed in one or more cells. 'Ec' is a droplet in which only *E. coli* was enclosed in one or more cells. 'Pm + Ec' is a droplet in which *P. mexicana* WO24 and *E. coli* were enclosed in one or more cells of each microorganism.

(a)

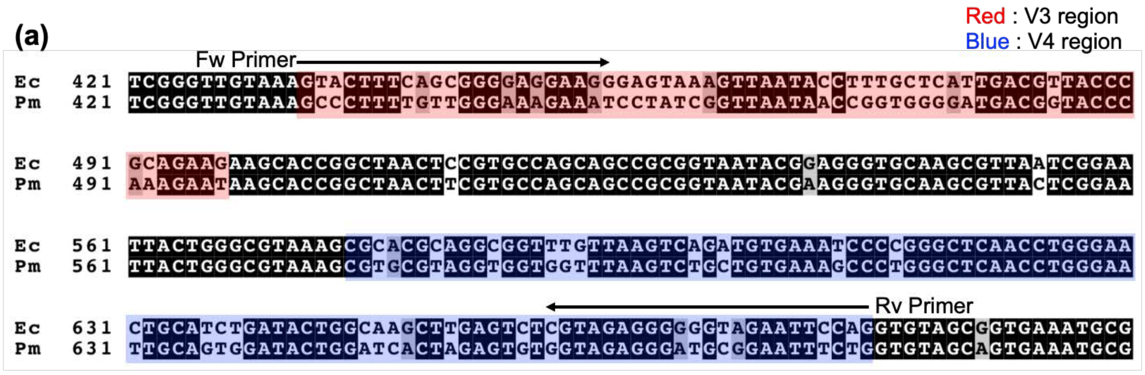

(b)

Primer names	Sequences (5'-3')
E. coli_V3-V4_Fw	GTACTTTCAGCGGGGAGGAAGG
E. coli_V3-V4_Rv	CTGGAATTCTACCCCCCTCTACG

(c)

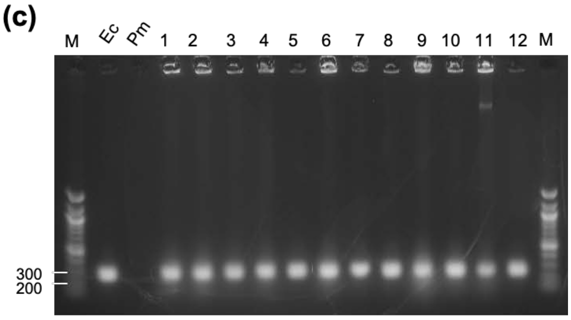

Figure S4 | Colony direct PCR for the V3-V4 region of 16S rDNA.

(a) DNA sequence alignment analysis of the V3-V4 region of 16S rDNA of *E. coli* and *P. mexicana* WO24. (b) PCR primers. (c) Agarose gel electrophoresis of PCR products.

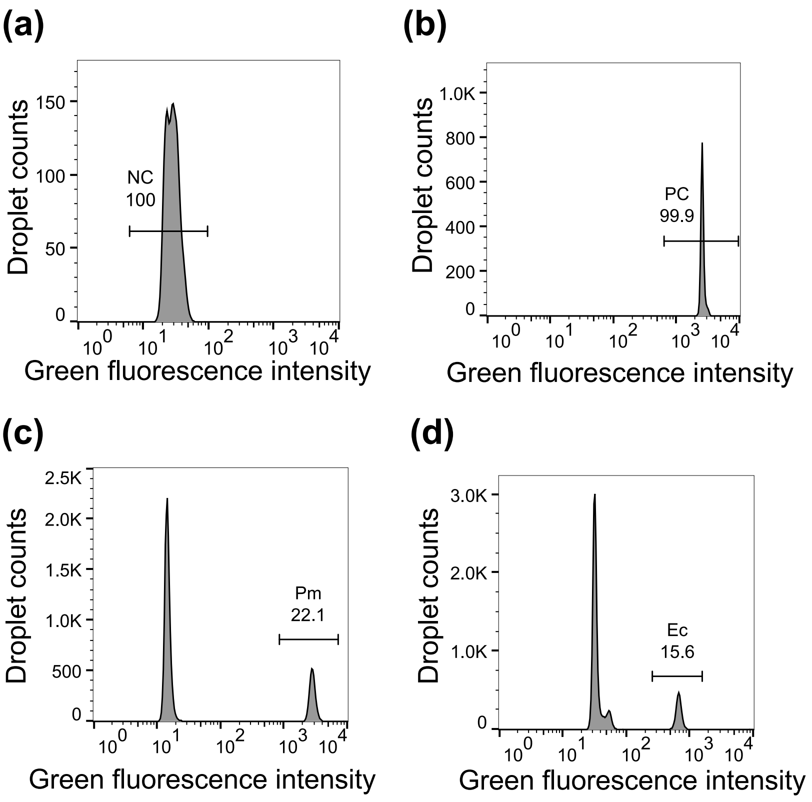

Figure S5 | FADS histograms of droplet analysis.

(a) Negative control sample contained only the substrate in medium. (b) Positive control sample contained droplet mixed with substrate and enzyme (Trypsin). (c) and (d) *P. mexicana* and *E. coli* cultured with fluorescent substrates in the WODL for 24 hours, respectively.

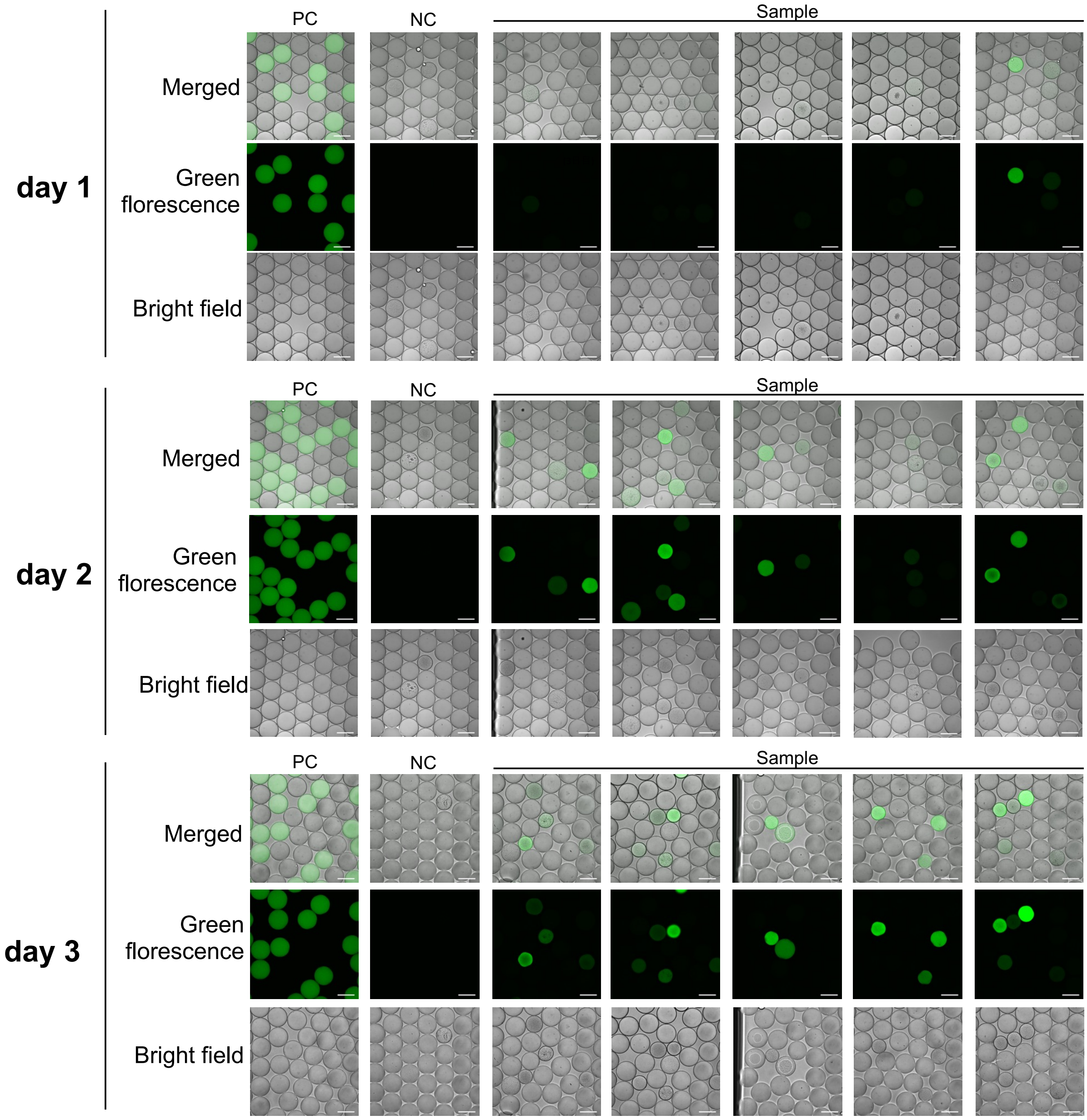

Figure S6 | Microscopic images of cultivated WODL for screening.

PC indicates positive control that had droplets mixed with enzyme (Trypsin) and droplets without enzyme. NC indicates negative control that is droplets cultured environmental microorganisms without substrate. The five sample images were taken at random on days 1 to 3. Images of sample were obtained randomly. Scale bar represents 100 µm.

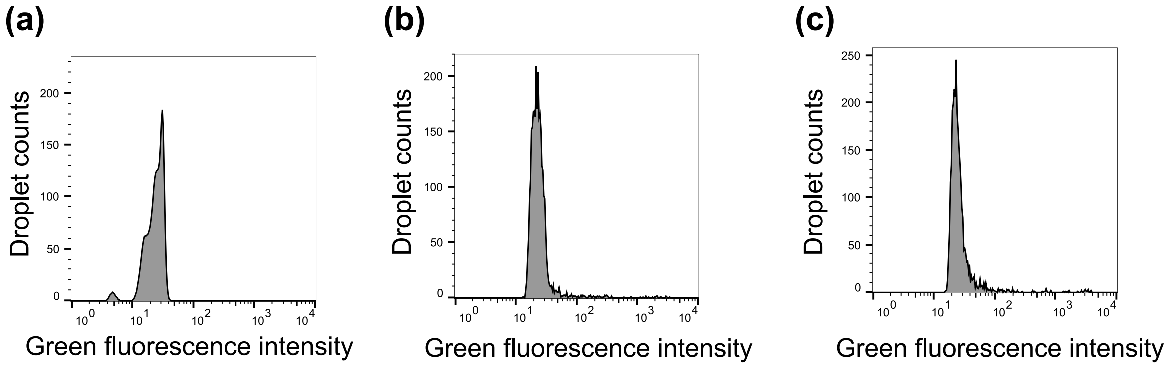

Figure S7 | FADS histograms of droplet analysis for screening of environmental microorganisms.

(a) Droplets encapsulating environmental microbes were analyzed for green fluorescence by FADS at each time point: (a) immediately after encapsulation, (b) after one day of cultivation, and (c) after two days of cultivation.

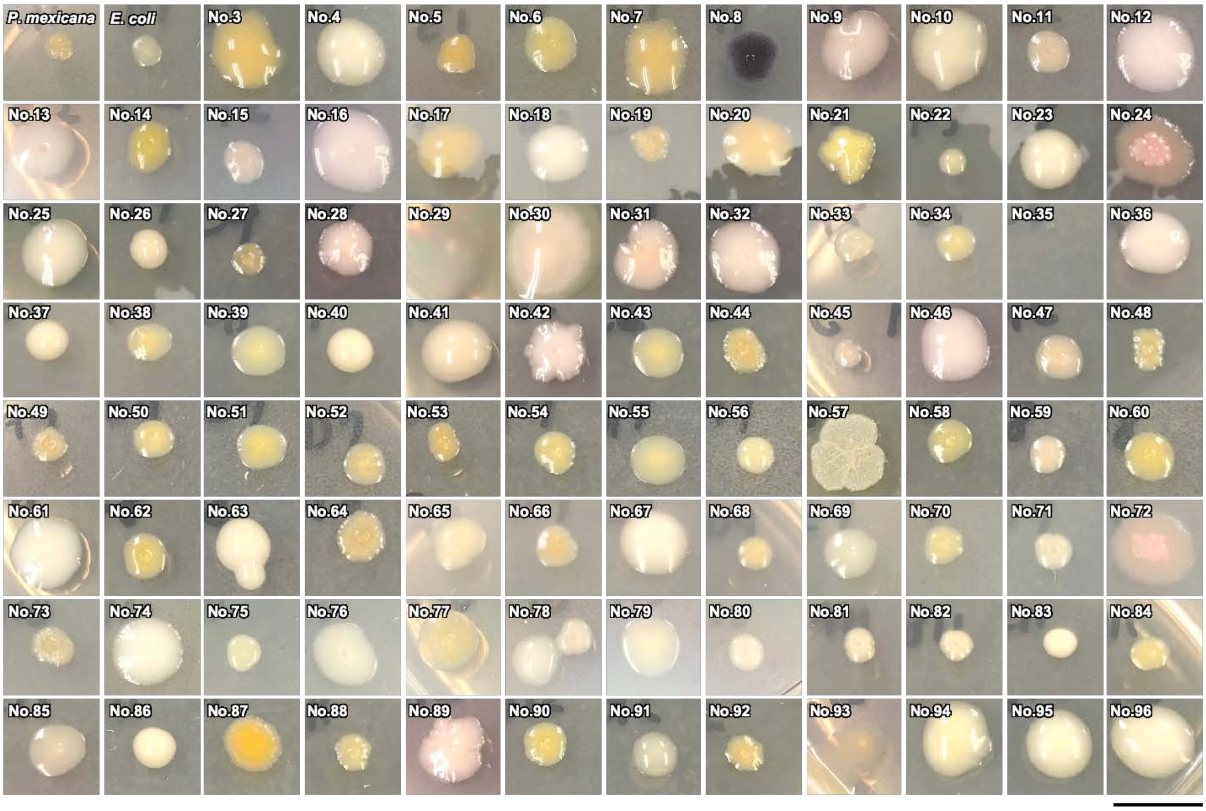

Figure S8 | Colony morphology of microorganisms isolated in this study.

When culturing in microplates, the bacteria were inoculated into replica plates and the morphology after 4 days of incubation at 25°C was documented and shown below the indicated numbers of strains. 1: *P. mexicana* WO24, No. 2: *E. coli*, No. 3–96: environmental microorganisms, Scale bar: 1 cm.

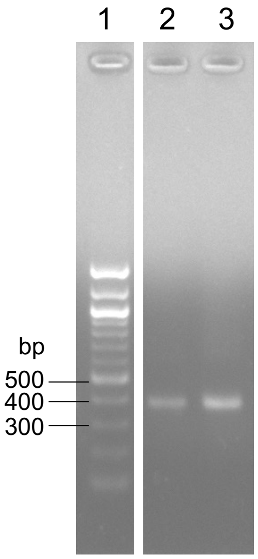

Lane No.	Sample name	Conc. (ng/μl)
2	Post-droplet cultivation	63.041
3	Post-sorting	89.337

Figure S9 | Agarose-gel electrophoresis of an ante-sequencing 16S rDNA sample.
1.5% agarose gel was used and 2 μl of amplified and purified 16S rDNA fragments were loaded to the lanes. Lane 1: 100 bp DNA Ladder (New England Biolabs, Japan); lane 2: post-droplet cultivation sample; lane 3: post-sorting sample

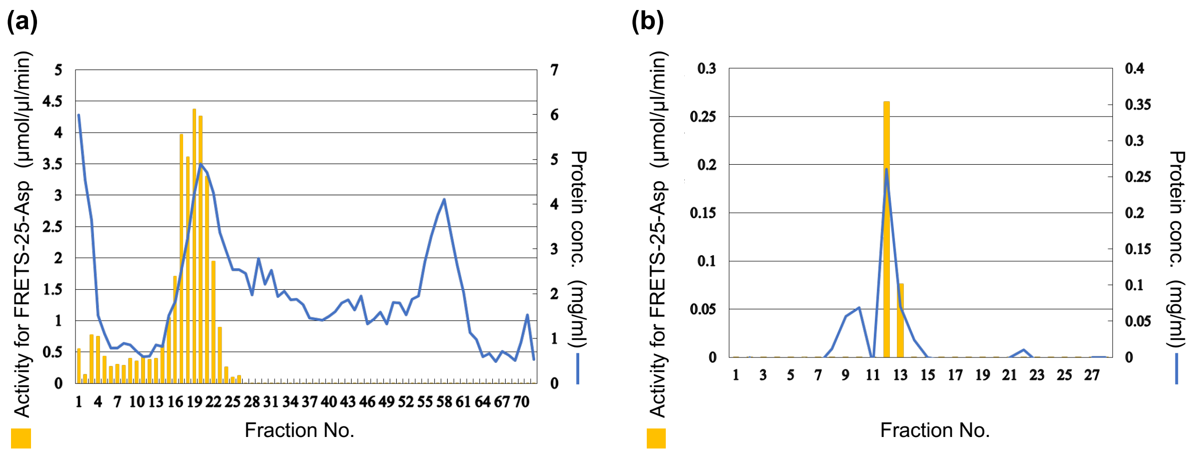

Figure S10 | Purification of Asp-specific endopeptidase from *L. soli*.

(A) Anion exchange chromatogram. (B) Gel filtration chromatogram. FRETS-25 aa series FRETS-25-Asp was used as a substrate for measurement of Asp-specific activity.

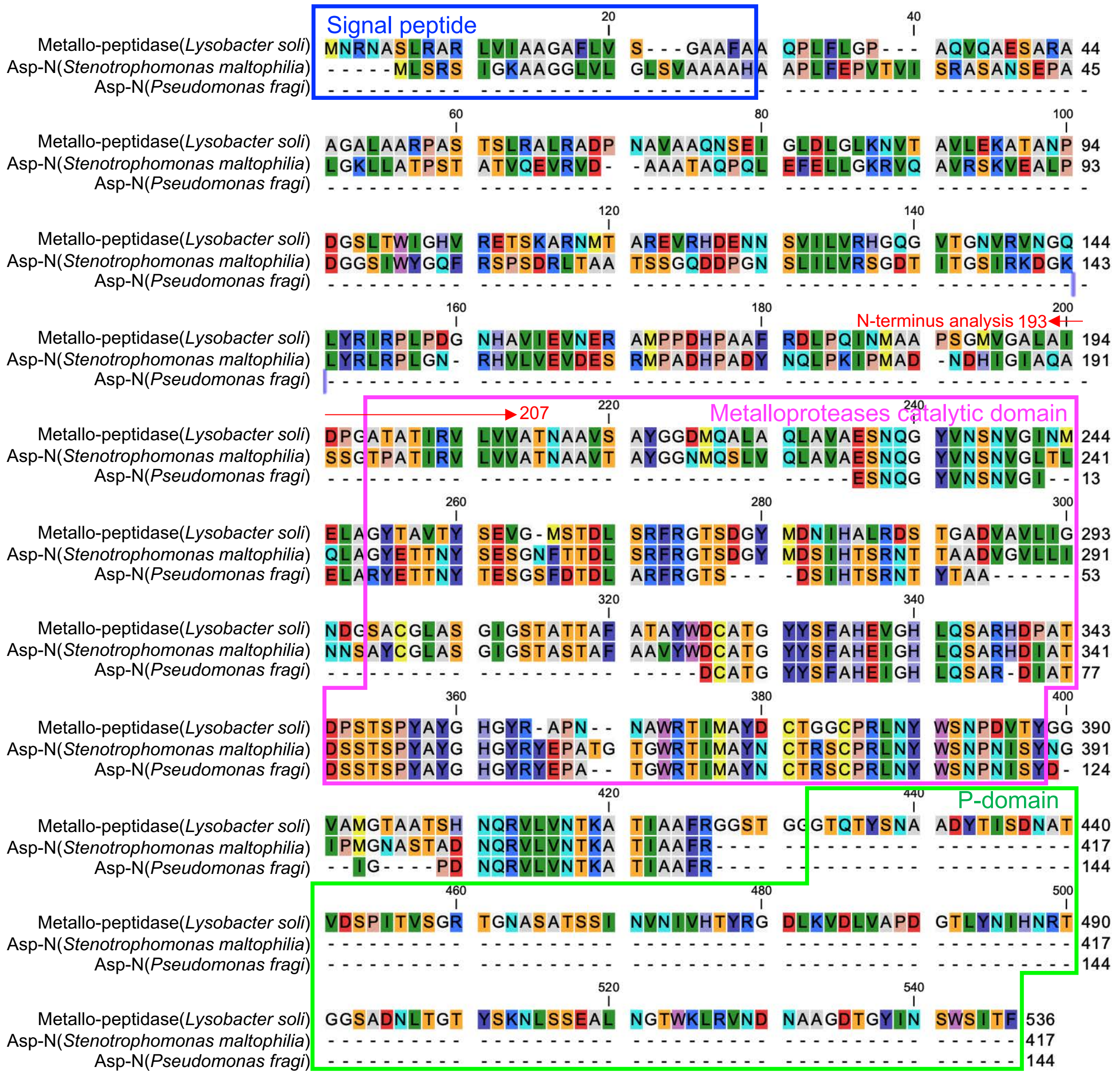

Figure S11 | Alignment analysis of two Asp-Ns and metallo-peptidase from *L. soli.*

The two Asp-N sequences were obtained by BLAST analysis in the Swiss-prot database using the amino acid sequence of metallo-peptidase from *L. soli* as the query sequence. The accession numbers of UniProtKB for SmAsp-N and PfAsp-N are B2FQP3 and Q9R4J4, respectively. InterPro release 97.0 was used for secondary structure prediction.
